# Nocturnal Melatonin Deficiency in Colorectal Cancer: Independent Predictive Value Beyond Sleep Quality

**DOI:** 10.5152/tjg.2026.25808

**Published:** 2026-02-23

**Authors:** Ibrahim Durak, Busra Durak, Tolga Duzenli, Musa Yilmaz, Muhammed Kaya, Mustafa Sadecolak, Huseyin Koseoglu, Duygu Ozol

**Affiliations:** 1 Department of Gastroenterology, Hitit University Faculty of Medicine, Çorum, Türkiye; 2 Department of Pulmonary Disease, Çorum Hitit University Faculty of Medicine, Çorum, Türkiye; 3 Department of Medical Biochemistry and Clinical Biochemistry, Hitit University Faculty of Medicine, Çorum, Türkiye; 4 Department of Gastroenterology, Samsun University Faculty of Medicine, Samsun, Türkiye; 5 Department of Chest Diseases, University of Health Sciences, Sureyyapasa Training and Research Hospital, İstanbul, Türkiye

**Keywords:** Colorectal cancer, Melatonin, PSQI, Sleep quality

## Abstract

**Background/Aim:**

Melatonin is a cytoprotective hormone with antioxidant activity and also mediates regulatory effects that inhibit tumor proliferation and angiogenesis. This study aimed to evaluate serum melatonin levels and sleep quality in patients with newly diagnosed colorectal cancer (CRC) compared with controls and to investigate whether melatonin could serve as a potential biomarker.

**Materials and Methods:**

A total of 71 participants (36 CRC, 35 controls) were included. Blood samples were obtained between 01:00-02:00 am and serum melatonin was measured using a high-sensitivity (ELISA) Enzyme-Linked Immunosorbent Assay kit. Sleep quality was assessed with the Pittsburgh Sleep Quality Index (PSQI) and sleep apnea risk was determined by the STOP-Bang questionnaire. Group comparisons were performed, followed by multivariable logistic regression and receiver-operating characteristic analysis.

**Results:**

Colorectal cancer patients had significantly lower nocturnal serum melatonin concentrations (131.1 ± 35.5 vs. 194.8 ± 50.8 pg/mL, *P* < .001) and higher PSQI scores (5.6 ± 3.1 vs. 5.1 ± 3.0, *P* = .028). In multivariable analysis, melatonin remained the only independent predictor of CRC (OR = 0.952, 95% CI: 0.929-0.976, *P* < .001). Receiver-operating characteristic analysis identified a melatonin threshold of ~150 pg/mL, discriminating CRC patients from controls with 75% sensitivity and 91.4% specificity (AUC = 0.877).

**Conclusion:**

Although cancer patients and the control group showed comparable obstructive sleep apnea risk, sleep quality was poorer, and melatonin levels were significantly lower in the cancer group. Melatonin assessment can complement traditional risk stratification and may provide new insights into the interplay between circadian biology, sleep, and colorectal carcinogenesis.

**Main Points**

- Nocturnal serum melatonin levels were significantly lower in colorectal cancer (CRC) patients compared with healthy controls.
- Melatonin remained the only independent predictor of CRC in multivariable logistic regression analysis, whereas subjective sleep quality and demographic factors were not significant.
- Although CRC patients reported poorer sleep quality, subjective sleep measures did not independently predict cancer status.
- Melatonin assessment may serve as a biologically grounded marker of circadian disruption and could complement traditional risk stratification in CRC.

## Introduction

Colorectal cancer (CRC) is among the most common malignancies worldwide and represents a leading cause of cancer-related morbidity and mortality.^1^ Despite advances in screening and treatment, identifying novel biomarkers that may improve early detection and provide insight into disease mechanisms remains a major research priority.^2^ Increasing evidence has suggested a link between circadian rhythm disruption, sleep quality, and cancer risk, including CRC.^3^^,^^4^

Melatonin (N-acetyl-5-methoxytryptamine) is a hormone mainly secreted by the pineal gland in the brain, but also synthesized in other tissues like the retina, gut, and skin. Melatonin has a pivotal role in the regulation of circadian rhythms and sleep-wake cycles.^5^ Beyond its chronobiological functions, melatonin exhibits antioxidant, anti-inflammatory, immunomodulatory, antiproliferative, and pro-apoptotic properties, which may contribute to its potential oncostatic effects.^6^^,^^7^ Preclinical studies have shown that melatonin can inhibit proliferation, angiogenesis, and metastasis in CRC cell lines and animal models.^8^^,^^9^ In addition, epidemiological data indicate that exogenous melatonin use may be associated with a reduced risk of CRC development.^10^

Sleep disturbances and poor sleep quality are frequently reported in CRC patients, often associated with fatigue, impaired quality of life, and worse clinical outcomes.^11^^,^^12^ Moreover, obstructive sleep apnea (OSA), characterized by intermittent hypoxia and sleep fragmentation, has been linked to an increased risk of CRC in population-based cohorts.^13^^,^^14^ These findings highlight the importance of investigating the relationship between melatonin, sleep quality, and CRC.

Given this background, the present study aimed to evaluate serum melatonin levels in patients with CRC compared with healthy controls and to explore the association of sleep quality parameters with CRC. It was hypothesized that melatonin levels would be lower in CRC patients and that altered sleep quality might contribute to the disease phenotype.

## Materials and Methods

### Study Design and Participants

This prospective observational study included 36 consecutive patients with newly diagnosed CRC confirmed by colonoscopy and histopathology and 35 healthy controls. Participants were recruited from the gastroenterology outpatient clinics between March and June 2025. Inclusion criteria were age between 45 and 75 years and willingness to participate in the study. Exclusion criteria included a history of previous intra-abdominal surgery, chronic kidney, heart, or liver failure, or electrolyte imbalance; presence of known sleep disorders such as OSA or insomnia, or chronic respiratory diseases; family history of cancer; use of medications affecting melatonin metabolism (such as melatonin supplements, beta-blockers, corticosteroids, or antidepressants); history of irritable bowel syndrome or other inflammatory or structural colon diseases such as Crohn’s disease, ulcerative colitis, celiac disease, or diverticulitis; excessive alcohol or tobacco use; and working night shifts or recent circadian rhythm disturbances (e.g., jet lag). All patients and controls were excluded with a family history of CRC or advanced adenomatous polyps. This study was approved by the Hitit University Clinical Research Ethics Committee (Approval No: 2024-43, Date: July 10, 2024).

### Data Collection

Demographic and clinical data, including age, sex, body mass index (BMI), smoking status, and comorbidities/regular drug use, were recorded for all participants. Sleep quality was assessed using the Pittsburgh Sleep Quality Index (PSQI), and sleep apnea risk was evaluated using the STOP-Bang questionnaire. The PSQI is a standardized questionnaire used to assess a person’s sleep quality over the previous month. It is widely used in clinical practice and sleep research to identify sleep problems and monitor treatment outcomes. It includes 19 items grouped into 7 components such as sleep efficiency, disturbances, and daytime dysfunction. Each component is scored from 0 to 3, with higher scores indicating worse sleep. The 7 component scores are summed to produce a global PSQI score ranging from 0 to 21. A global score >5 typically indicates poor sleep quality. The STOP-Bang questionnaire is a simple, validated screening tool used to identify individuals at risk for OSA. It includes 8 yes/no questions covering Snoring, Tiredness, Observed apneas, High blood pressure, BMI, Age, Neck circumference, and Gender. Each “yes” answer scores 1 point, for a total score ranging from 0 to 8. Higher scores indicate a greater likelihood of moderate-to-severe OSA. A score of 0-2 = low risk, 3-4 = intermediate risk, and 5-8 = high risk for OSA.

### Blood Sampling and Melatonin Measurement

All blood samples for melatonin measurement were collected on the night following diagnostic colonoscopy, during nocturnal sleep between 01:00-02:00 am, in a dark and quiet hospital room to minimize circadian variability. This time window corresponds to the well-established nocturnal peak of endogenous melatonin secretion as reported in circadian rhythm literature.^15^^-^^17^ Serum was separated by centrifugation and stored at −80°C until analysis. Melatonin concentrations were determined using a commercially available Human MT (Melatonin) ELISA Kit (E-EL-H2016, Elabscience, Houston, TX, USA) according to the manufacturer’s protocol.

### Sample Size and Power Calculation

Sample size estimation was based on a previous study reporting markedly lower nocturnal melatonin levels in advanced solid tumor patients compared with healthy controls (12.3 ± 3.5 pg/mL vs. 38.7 ± 6.4 pg/mL; cancer n = 45, control n = 20).^18^ Although the calculated effect size from these values is extremely large (Cohen’s *d* ≈ 5), a more conservative approach was adopted to account for biological variability and to avoid overestimating power. Therefore, a large effect size (*d* = 0.8) was assumed for planning. Using G*Power 3.1 (Heinrich-Heine-Universität Düsseldorf; Düsseldorf, Germany) with *α* = 0.05 and 80% power, at least 25 participants per group were required. Accordingly, the study aimed to include ≥25 CRC patients and ≥25 healthy controls, and the final sample size met this target.

### Statistical Analysis

Statistical analyses were performed using SPSS, version 21 (IBM SPSS Corp.; Armonk, NY, USA). Continuous variables are expressed as mean ± standard deviation or median interquartile range (IQR), and categorical variables as frequency and percentage. Between-group comparisons were made using Student’s *t*-test or Mann–Whitney *U*-test for continuous variables and chi-square or Fisher’s exact test for categorical variables. Logistic regression analysis was conducted with CRC diagnosis as the dependent variable and melatonin levels (and other covariates where applicable) as independent variables. Variables with *P* < .10 in univariate analysis (smoking and diabetes mellitus) were additionally tested in a multivariable logistic regression model. However, based on the events per variable principle and absence of independent significance, the final model was presented using melatonin, age, sex, and PSQI. Receiver operating characteristic (ROC) analysis was performed to evaluate the discriminatory power of melatonin. A *P*-value <.05 was considered statistically significant.

## Results

A total of 71 participants were included in the study, of whom 36 were newly diagnosed with CRC and 35 served as controls. The demographic and categorical variables of the groups are summarized in Table 1. Smoking habit, STOP-Bang classification, and distribution of comorbidities were similar between groups, although diabetes mellitus was more common in CRC patients compared with controls (22.2% vs. 8.6%, *P* = .085). Hypertension and coronary artery disease were also more frequent in the CRC group, but the differences did not reach statistical significance.

Continuous clinical, sleep-related, and biochemical parameters are presented in Table 2. Colorectal cancer patients tended to be older than controls (63.6 ± 6.8 vs. 58.5 ± 2.9 years, *P* = .179), although the difference was not statistically significant. Body mass index was comparable between the groups. Pittsburgh Sleep Quality Index scores were significantly higher in CRC patients compared with controls (5.6 ± 3.1 vs. 5.1 ± 3.0, *P* = .028), indicating poorer subjective sleep quality. Importantly, nocturnal serum melatonin levels were markedly lower in the CRC group than in healthy participants (131.1 ± 35.5 vs. 194.8 ± 50.8 pg/mL, *P* < .001).

Melatonin levels were further analyzed according to CRC tumor stages. Eight patients were Stage 1, 14 Stage 2 (2A+2B combined), 5 Stage 3, and 9 Stage 4. The median (IQR) melatonin values were 135.19 (127.28-148.36) pg/mL in Stage 1, 146.37 (105.39-157.02) pg/mL in Stage 2, 112.32 (93.43-146.64) pg/mL in Stage 3, and 116.65 (109.13-120.73) pg/mL in Stage 4. No statistically significant difference was observed between individual stages (*P* > .05). When grouped as early stage (Stage 1-3) vs. late stage (Stage 4), median melatonin levels were 140.35 pg/mL (IQR: 107.14-155.81) vs. 116.65 pg/mL (IQR: 109.13-120.73), respectively (*P* = .195).

In multivariable logistic regression analysis including age, gender, PSQI, and melatonin levels (Table 3), serum melatonin was found to be an independent predictor of CRC (β = −0.049, OR = 0.952, 95% CI: 0.929-0.976, *P* < .001). Neither age (OR = 1.055, *P* = .353), gender (OR = 0.318, *P* = .136), nor PSQI (OR = 1.233, *P* = .123) were independently associated with CRC.

**Figure 1.**
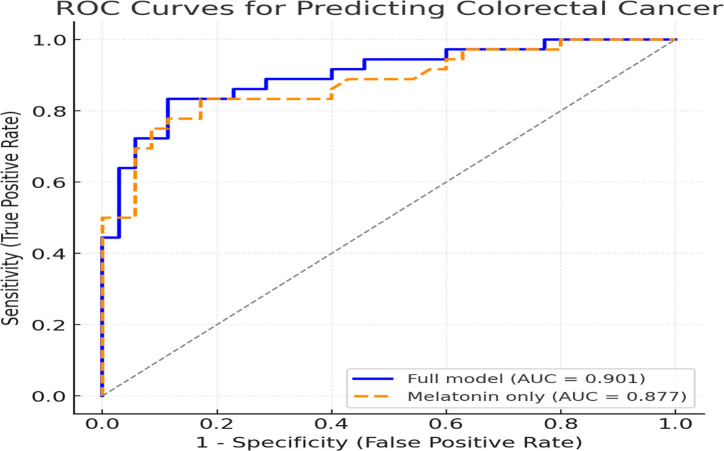
Comparison of receiver operating characteristic (ROC) curves for predicting colorectal cancer using melatonin alone (AUC = 0.877) vs. the full multivariable logistic regression model (AUC = 0.901).

The diagnostic performance of melatonin and the multivariable model was evaluated using ROC analysis. A melatonin threshold of approximately 150 pg/mL discriminated CRC patients from controls with 75% sensitivity and 91.4% specificity (AUC = 0.877). As illustrated in Figure 1, melatonin alone demonstrated good discriminative ability, while the full logistic regression model incorporating melatonin, age, gender, PSQI, and hypertension achieved an even higher accuracy (AUC = 0.901).

## Discussion

In this study, it was demonstrated that serum melatonin concentrations were significantly lower in patients with newly diagnosed CRC compared to healthy controls. Importantly, melatonin remained the only independent predictor of CRC in multivariable logistic regression analysis including age, gender, smoking history, and PSQI. Receiver-operating characteristic analysis further supported its diagnostic value, showing good discriminative ability for CRC, which improved modestly when combined with clinical parameters. These findings suggest that melatonin depletion may be involved in colorectal carcinogenesis and highlight its potential as a non-invasive biomarker.

Melatonin is a pleiotropic indoleamine with well-established antioxidant, cytoprotective, and immunomodulatory actions; however, emerging evidence indicates that its physiological role extends beyond circadian regulation to include direct effects on gut barrier integrity and host–microbiota interactions. Experimental data show that melatonin protects intestinal epithelial tight-junction structure, decreases gut permeability, and mitigates mucosal injury under inflammatory or toxic conditions.^19^^-^^21^ Additionally, melatonin has been implicated in modulating gut microbiota composition and host–microbiota signaling, with studies demonstrating that certain human gut bacteria respond to melatonin and exhibit melatonin-dependent circadian-like behavior.^22^ Dysbiosis, particularly microbial patterns linked to colorectal carcinogenesis, including the overgrowth of Fusobacterium nucleatum, may be shaped by disrupted melatonin signaling, offering a mechanistic bridge between circadian dysregulation, microbiota imbalance, and CRC risk.^23^^,^^24^

In the study, nocturnal serum melatonin levels were significantly lower in patients with CRC compared with controls. This finding is consistent with previous reports. Khoory and Stemme first described reduced plasma melatonin concentrations in CRC patients,^25^ and Kos-Kudła et al subsequently confirmed attenuated nocturnal secretion and blunted circadian amplitude in this population.^26^ Moreover, Vician et al demonstrated that melatonin levels increased after surgical tumor removal, suggesting that the tumor itself may suppress pineal secretion.^27^ At the molecular level, renewed attention to melatonin has emphasized its pleiotropic anti-cancer effects: beyond circadian signaling, melatonin acts as a direct free-radical scavenger, upregulates antioxidant enzymes, stabilizes mitochondrial function, and reduces DNA damage and apoptotic susceptibility in non-malignant tissues—properties that plausibly protect against tumor initiation in the gut mucosa.^28^

Unlike these earlier investigations, which mostly relied on radioimmunoassay with limited sensitivity and variable sampling schedules, the study used a high-sensitivity ELISA kit and strictly standardized blood collection between 01:00 and 02:00 am, while excluding patients with known sleep or respiratory disorders. These methodological strengths provide more robust evidence that melatonin reduction is a reproducible feature of CRC. The observed decrease may represent either a contributing factor to carcinogenesis through impaired circadian regulation, enhanced oxidative stress, and inflammation or a consequence of tumor-related suppression of melatonin synthesis.^27^^,^^29^ Given the cross-sectional design, causality cannot be determined, and further longitudinal research is warranted.

Although poor sleep quality disrupts circadian rhythms, impairs immune surveillance, and increases oxidative stress—factors that may contribute to cancer initiation and progression—the role of sleep quality as a causal factor in CRC, or merely a consequence of the disease, remains unclear. Night-shift work, which causes circadian disruption and is classified by the International Agency for Research on Cancer as probably carcinogenic to humans, is also considered a risk factor for CRC. In the study, PSQI scores were higher in CRC patients than in controls, consistent with prior observations. Studies and meta-analyses continue to link altered sleep patterns—both short and long sleep duration, fragmented sleep, and sleep-disordered breathing—to higher incidence of colorectal neoplasms, suggesting that disturbed sleep is a modifiable risk factor deserving prospective study. On the other hand, CRC patients often experience disrupted sleep due to cancer-related pain, gastrointestinal symptoms, or psychological distress, suggesting that poor sleep may also be secondary to the disease process.^30^^,^^31^ Epidemiological studies have linked both insomnia and chronic sleep disturbances to increased cancer risk, possibly through these mechanisms with additional systemic inflammation and hormonal dysregulation surveillance.^32^^,^^33^ However, PSQI did not remain an independent predictor in the multivariable analysis. This may reflect that melatonin provides a more direct biological measure of circadian disruption, while subjective questionnaires are susceptible to recall bias and psychosocial influences. Alternatively, the lack of statistical significance could be explained by the limited sample size and reduced power in multivariable modeling.

Another important finding of the study is that melatonin remained an independent predictor of CRC, whereas PSQI did not. This discrepancy may be explained by fundamental differences between subjective sleep questionnaires and objective hormonal markers. Pittsburgh Sleep Quality Index captures patients’ perception of sleep quality, which is influenced by psychological status, comorbid conditions, and recall bias.^34^^,^^35^ In contrast, melatonin reflects a direct biological output of the circadian system and integrates the effects of light exposure, sleep-wake rhythm, and neuroendocrine regulation.^36^^,^^37^ Therefore, reduced melatonin concentrations may serve as a more proximal marker of circadian disruption than self-reported sleep quality. The results support the hypothesis that melatonin, rather than subjective sleep indices, could provide a more reliable biomarker for circadian dysregulation in the context of CRC.

Several variables traditionally associated with CRC risk did not reach statistical significance in the study. Age, sex, and BMI are well-established epidemiological risk factors for CRC, yet no significant differences were observed between the groups. This is likely due to the relatively narrow age range of the participants and the limited sample size, which reduce the statistical power to detect modest effects.^2^^,^^38^ Common comorbidities such as hypertension, diabetes mellitus, and coronary artery disease also showed higher proportions in CRC patients but without reaching statistical significance, again most likely due to sample size limitations and low prevalence in controls.^39^

Although STOP-Bang categories did not differ significantly between groups, it is noteworthy that a subset of individuals in the control group fell into the high-risk category. Because STOP-Bang is a screening tool rather than a diagnostic test, elevated scores can occur in otherwise healthy individuals due to factors such as age, male sex, or neck circumference. However, polysomnography was not performed in the control group; therefore, unrecognized or subclinical OSA cannot be completely excluded. This potential misclassification may have attenuated the true association between sleep-related breathing disturbances and CRC and may partially explain why PSQI did not remain an independent predictor in the multivariable model. Even though Fisher’s exact test did not demonstrate a statistically significant difference (*P *≈ .19), the possibility of overlap between groups underscores the need for future studies incorporating objective sleep assessments. Taken together, these null findings highlight both the strengths of the standardized design and the constraints imposed by a modest sample size, underscoring the need for larger multicenter studies to validate these associations.

Although the stage-based comparison did not reach statistical significance, melatonin levels showed a downward trend from early to advanced CRC, with the largest difference observed between Stage 1 and Stage 4. This pattern may suggest that melatonin suppression is more pronounced in advanced disease, potentially reflecting greater circadian disruption with increasing tumor burden. However, subgroup numbers were small, especially in Stage 3, and variability may have limited statistical power. Larger studies with balanced stage distribution are needed to clarify whether melatonin could serve as an early biological signal or prognostic indicator across CRC stages.

From a clinical perspective, nocturnal serum melatonin measurement may serve as a complementary biomarker in CRC. Although it is not yet suitable as an independent screening tool, its significant reduction in CRC patients suggests that melatonin could enhance conventional risk-stratification approaches when used alongside established screening methods such as fecal immunochemical test or colonoscopy. Individuals with markedly low melatonin levels might be considered for closer follow-up or earlier endoscopic assessment. This aligns with recent evidence highlighting the growing interest in biomarker-based and data-driven risk prediction approaches in CRC, including genomic and transcriptomic marker studies as well as machine-learning-assisted classification models.^40^^-^^42^ Future large-scale prospective studies are required to establish threshold values and to determine whether melatonin can be integrated into CRC screening or prognostic frameworks.

This study has several limitations that should be acknowledged. First, its cross-sectional design does not allow for causal inference regarding the relationship between melatonin, sleep quality, and CRC. Second, the relatively small sample size limits statistical power, particularly in the analysis of comorbid conditions and in multivariable models, where some associations may have failed to reach significance due to insufficient numbers. Third, CRC-related symptoms such as abdominal discomfort, altered bowel habits, or cancer-associated anxiety may independently influence sleep quality and melatonin secretion, and although patients were evaluated at diagnosis before treatment initiation, the impact of symptom burden cannot be fully excluded. Finally, as a single-center study, the generalizability of the findings may be restricted, and replication in larger, multicenter cohorts is warranted. On the other hand, the narrow age range of the study population increases the reliability of both sleep-related data and melatonin levels.

In conclusion, the recent work has deepened the understanding of the complex links among sleep, melatonin, and CRC. In the study, although cancer patients and the control group were similar in terms of sleep quality and OSA risk, melatonin levels were found to be significantly lower in the cancer group. From a clinical perspective, melatonin assessment could complement traditional risk stratification and may provide new insights into the interplay between circadian biology, sleep, and colorectal carcinogenesis. Sleep quality should be routinely evaluated in patients with CRC, with particular attention to prolonged sleep latency and frequent sleep fragmentation. Sleep hygiene education should be provided, and in selected cases, targeted treatment—including melatonin supplementation—may be considered. Nevertheless, larger longitudinal studies are needed to confirm these findings and to clarify whether melatonin could eventually be incorporated into CRC screening or prognostic frameworks.

## Figures and Tables

**Table 1.** Demographic and categorical variables between colorectal cancer and control groups

**Variable**	**Colorectal Cancer (n = 36)**	**Control (n = 35)**	***P***
Gender (male, %)^1^	58.3	62.9	.883
Smoking (% never/former/current)^2^	69.4/22.2/8.3	60.0/40.0/0.0	.088
STOP-Bang category (% low/moderate/high)^2^	16.7/38.9/44.4	34.3/37.1/28.6	.194
Comorbidities^1^			
Diabetes mellitus (%)	8 (22.2)	2 (5.7)	.085
Hypertension (%)	9 (25)	4 (11.4)	.241
Coronary artery disease (%)	6 (16.7)	3 (8.6)	.478

^1^Chi-square.

^2^Fisher’s exact test.

BMI, body mass index; PSQI, Pittsburgh Sleep Quality Index; STOP-Bang, Snoring–Tiredness–Observed apnea–high Blood pressure–BMI–Age–Neck circumference–Gender.

**Table 2.** Comparison of Continuous Clinical, Sleep, and Melatonin Parameters Between Colorectal Cancer and Control Groups

**Variable**	**Colorectal Cancer (n = 36)**	**Control (n = 35)**	***P***
Age (years)^1^	63.6 ± 6.7	61.6 ± 5.2	.179
BMI (kg/m^2^)^1^	26.1 ± 4.6	26.4 ± 4.1	.751
PSQI score^2^	6 ± 3	4 ± 3	**.027**
Melatonin (pg/mL)^1^	131.1 ± 35.5	194.8 ± 50.8	**<.001**

^1^*t* -test, mean ± SD

^2^Mann–Whitney *U*-test, median ± IQR.

BMI, body mass index; PSQI, Pittsburgh Sleep Quality Index.

**Table 3.** Logistic Regression Analysis for Predictors of Colorectal Cancer

**Variable**	**β (Coef.)**	**OR (Exp(β))**	**95% CI for OR**	***P***
Age	+0.054	1.055	0.942-1.181	.353
Melatonin	−0.049	0.952	0.929-0.976	**<.001**
PSQI	+0.209	1.233	0.945-1.609	.123
Gender	−1.144	0.318	0.071-1.433	.136

BMI, body mass index; PSQI, Pittsburgh Sleep Quality Index; OR, odds ratio.

Significance of bold values *P* < 0.05.
